# Preferential Coupling of Dopamine D_2S_ and D_2L_ Receptor Isoforms with G_i1_ and G_i2_ Proteins—In Silico Study

**DOI:** 10.3390/ijms21020436

**Published:** 2020-01-09

**Authors:** Justyna Żuk, Damian Bartuzi, Dariusz Matosiuk, Agnieszka A. Kaczor

**Affiliations:** 1 Department of Synthesis and Chemical Technology of Pharmaceutical Substances with Computer Modeling Laboratory, Faculty of Pharmacy, Medical University of Lublin, 4A Chodzki St., PL-20093 Lublin, Poland; j.siudem@gmail.com (J.Ż.); damian.bartuzi@gmail.com (D.B.); dariusz.matosiuk@umlub.pl (D.M.); 2 School of Pharmacy, University of Eastern Finland, Yliopistonranta 1, P.O. Box 1627, FI-70211 Kuopio, Finland

**Keywords:** dopamine D_2_ receptor, GPCRs, molecular dynamics, molecular switches, principal component analysis

## Abstract

The dopamine D_2_ receptor belongs to rhodopsin-like G protein-coupled receptors (GPCRs) and it is an important molecular target for the treatment of many disorders, including schizophrenia and Parkinson’s disease. Here, computational methods were used to construct the full models of the dopamine D_2_ receptor short (D_2S_) and long (D_2L_) isoforms (differing with 29 amino acids insertion in the third intracellular loop, ICL3) and to study their coupling with G_i1_ and G_i2_ proteins. It was found that the D_2L_ isoform preferentially couples with the G_i2_ protein and D_2S_ isoform with the G_i1_ protein, which is in accordance with experimental data. Our findings give mechanistic insight into the interplay between isoforms of dopamine D_2_ receptors and G_i_ proteins subtypes, which is important to understand signaling by these receptors and their mediation by pharmaceuticals, in particular psychotic and antipsychotic agents.

## 1. Introduction

Dopamine receptors belong to rhodopsin-like G protein-coupled receptors (GPCRs) and share the molecular architecture typical for this family of proteins. They consist of seven transmembrane (TM) helices, which are connected by three extracellular and three intracellular loops (ECL1, ECL2, ECL3, and ICL1, ICL2, ICL3, respectively). Dopamine receptors are divided into D_1_-like (D_1_ and D_5_ receptors) and D_2_-like (D_2_, D_3_, and D_4_ receptors) subfamilies based on their activation or inhibition of adenylyl cyclase. These aminergic GPCRs are involved in regulation of many physiological conditions, ranging from voluntary movement and reward to hormonal regulation and hypertension [1], and thus they are important molecular targets for the treatment of a number of diseases: Parkinson’s disease, restless legs syndrome, sexual dysfunction, dementia, depression, bipolar disorder, Huntington’s disease, and schizophrenia [2]. Among dopamine receptor subtypes, D_2_ is the most widely explored in medicinal chemistry, being a target for antipsychotics, drugs against Parkinson’s disease, and many other drugs.

The dopamine D_2_ receptor inhibits adenylyl cyclase as it is coupled to G_i/o_ protein. Two alternatively spliced variants are produced from the D_2_ receptor gene and code for the D_2L_ and D_2S_ isoforms, which are 444 and 415 amino acids in length, respectively [3]. These isoforms share comparable pharmacological features and are expressed in the same cell types, with a ratio that usually favors the expression of the longer isoform [3]. The D_2S_, however, is dominant in the cell bodies and projection axons of the dopaminergic cell groups of the mesencephalon and hypothalamus, while the D_2L_ is more strongly expressed by neurons in the striatum and nucleus accumbens, brain structures targeted by dopaminergic fibers [4]. The D_2L_ isoform differs from D_2S_ by the insertion of 29 amino acids in the ICL3 of the receptor. This loop is engaged in the coupling of the receptor to various intracellular partners. The available data show that the D_2_ isoforms have differentiated affinities to different G proteins, implying that these receptors might have different roles in vivo [3]. For instance, it was found that dopamine D_2S_ and D_2L_ receptors may differentially contribute to the actions of antipsychotic and psychotic agents in mice [5].

In 1993, Montmayeur et al. showed that the 29 amino acids insertion present in D_2L_ allows it to interact specifically with G_i2_ [6]. Moreover, in vivo studies indicate that the D_2L_ isoform is preferentially coupled to G_i2_ and D_2S_ prefers G_i1_ over G_i2_ [7]. In 1994, Sengoles performed a study with the mutant G_iα_ proteins and found that the D_2L_ isoform signaled through the G_i3_ protein and D_2S_ through G_i2_ [8]. In 2004, Sengoles et al. created two-point mutations of the D_2S_ receptor in ICL3 by random mutagenesis (R233G and A234T) [9]. The mutant receptors exhibited a change in the G_i_ subtype coupling preference in comparison to a native D_2S_ receptor, suggesting the importance of the ICL3 sequence for G protein interaction. Therefore, while available data gives some premises, details of preferential coupling of the isoforms with G_i_ protein subtypes remain unclear.

The interaction of D_2S_ or D_2L_ isoforms with an agonist results in the process of receptor activation and subsequent binding with G protein. The outward movement of TM6 is the most pronounced conformational change on the cytoplasmic side of the receptor occurring during activation as reported for β_2_ adrenergic receptor [10]. This change is assisted by the outward movement of TM5 and a slight inward adjustment in the position of TM3 and TM7 to accommodate space for the binding of G protein [10]. The mechanisms of activation are, however, common in the whole family A of GPCRs.

The process of signal transmission from the agonist binding site on the extracellular part of the receptor to its intracellular part is enabled by molecular switches [11]. There are four widely approved molecular switches believed to take part in activation of class A GPCRs: two switches involving movements of specific side chains including (1) the W6.48 tryptophan toggle switch (the CWxP motif in TM6) accompanied by a transmission switch involving neighboring amino acids and (2) the Y7.53 tyrosine toggle switch (the NPxxY motif in TM7); as well as two other switches operating by breaking of bonds/interactions linking TMs: (3) the ionic lock involving helices TM3 and TM6, and (4) the 3–7 lock linking helices TM3 and TM7 (residue numbers according to Ballesteros–Weinstein nomenclature [12]) [11,13].

In spite of about 350 GPCR crystal structures including 85 active structures [14] in the Protein Data Bank, the full experimentally solved structures of receptors with long and flexible ICL3 are not yet available. Due to limitations of experimental methods, at present, such structures can be only obtained using molecular modeling approaches as it has been recently done, e.g., for the human serotonin 5-HT_2A_ receptor [15]. In this work, we aimed to construct complete models of D_2S_ and D_2L_ isoforms in complex with dopamine and both G_i1_ and G_i2_ proteins and, subsequently, to examine possible G protein coupling preferences of these isoforms and the receptor activation/deactivation hallmarks upon agonist binding and G protein coupling with molecular dynamics (MD) techniques.

## 2. Results

### 2.1. Construction of the Models of D_2L_ and D_2S_ Receptors in Complex with G_i1_ and G_i2_ Proteins

**Figure 1:**
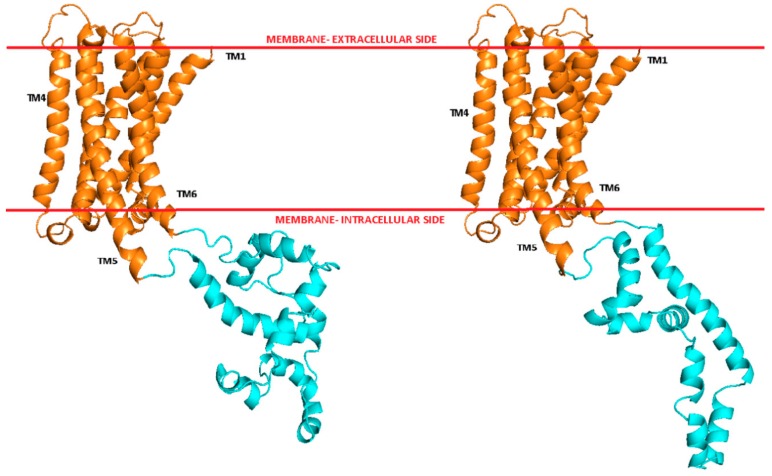
Models of transmembrane helices, TM, (orange) and ICL3 loop (cyan) of D_2L_ (left) and D_2S_ (right) receptors.

First, we constructed models of the studied receptors in active conformation: D_2L_ receptor in complex with G_i1_ protein (L1), D_2L_ receptor in complex with G_i2_ protein (L2), D_2S_ receptor in complex with G_i1_ protein (S1), and D_2S_ receptor in complex with G_i2_ protein (S2) using homology modeling with Modeller v.9.19 and Yasara tool for loop modeling. The attempts to model the ILC3 loop with Robetta [16] or I-Tasser [17] were not successful. The crystal structure of human β_2_-adrenergic receptor in complex with a heterotrimeric G_s_ protein (PDB ID: 3SN6 [18]) was used as a template for the helical bundle and G protein while the crystal structures of human dopamine D_2_, D_3_, and D_4_ receptors (PDB IDs: 6CM4 [19], 3PBL [20], and 5WIU [21], respectively) were used as templates for the extracellular loops. The sequence identity between the dopamine D_2_ receptor and β_2_adrenergic receptor is 37% and the sequence similarity 59%. The final models were selected from the generated populations of 100 models each (four final models from each population, 16 models in total). Figure 1 shows best-scored models of D_2S_ and D_2L_ receptors with the ICL3 loop. The transmembrane region of the receptors displays the features of the active conformation. The ICL3 loop connects the intracellular termini of TM5 and TM6. As predicted by the Predict Protein [22] online server, both the short and long variant of the loop are constituted by a few α-helical regions connected by disordered regions.

The best-scored receptor-G protein complexes were validated using ProCheck [23], Verify3D [24], and ERRAT [25], as well as by molecular docking. The results of validation of the 16 best-scored models are presented in Table 1. It can be seen that, according to the used tools, the obtained models are characterized by good quality. According to ProCheck, over 90% of residues in all the models fall into the favored region and only about 1% of amino acids are located in the outlier region. As visualized by the Ramachandran plots, no outliers were found in the transmembrane regions of the receptor models. Most outliers were glycine and proline residues located in loop regions of G proteins. Moreover, according to Verify3D, the final models had over 80%of residues that had an average 3D–1D scores ≥0.2 and, according to ERRAT, the overall quality factor in all chosen models exceeded 90.

### 2.2. Ligand Docking

**Figure 2:**
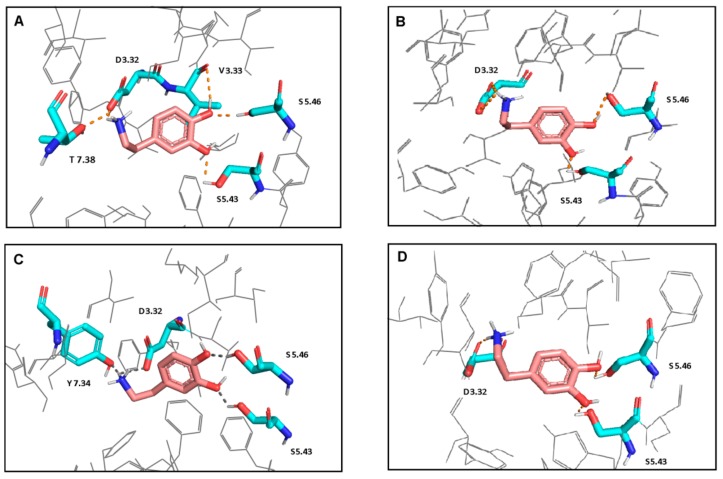
Interactions of dopamine with L1 (**A**), L2 (**B**), S1 (**C**), and S2 (**D**) models. Ligand is shown in stick representation with pink carbon atoms. Proteins are colored in gray and shown in wire representation. The interacting residues are shown in stick representation with blue nitrogen, red oxygen and cyan carbon atoms. Hydrogen bonds and salt bridges are shown as the orange dot- lines. Non-polar hydrogen atoms are not shown. Polar hydrogen atoms are shown only for the ligand and the interacting residues.

A natural agonist—dopamine—was docked with Molegro software to sixteen best-scored receptor-G protein complexes models. The final docking poses of the ligand to four models (Figure 2) were selected based on the scoring functions values, visual inspection and literature data. As typical for the orthosteric ligands of aminergic GPCRs, the protonatable nitrogen atom of dopamine forms a salt bridge with the carboxylic group of the D3.32 in TM3. Moreover, meta- and para hydroxyl groups of the catechol ring form hydrogen bonds with the side chains of S5.43 and S5.46 in TM5, respectively. In the case of L1 complex, an additional hydrogen bond interaction of the para-hydroxy group of dopamine with the main chain of V3.33 was found. In the case of L1 and S1 complexes, the hydrogen bond was also observed between the protonatable nitrogen atom of the ligand and the side chain of T7.38 or Y7.34, respectively.

### 2.3. Molecular Dynamics Simulations

**Figure 3:**
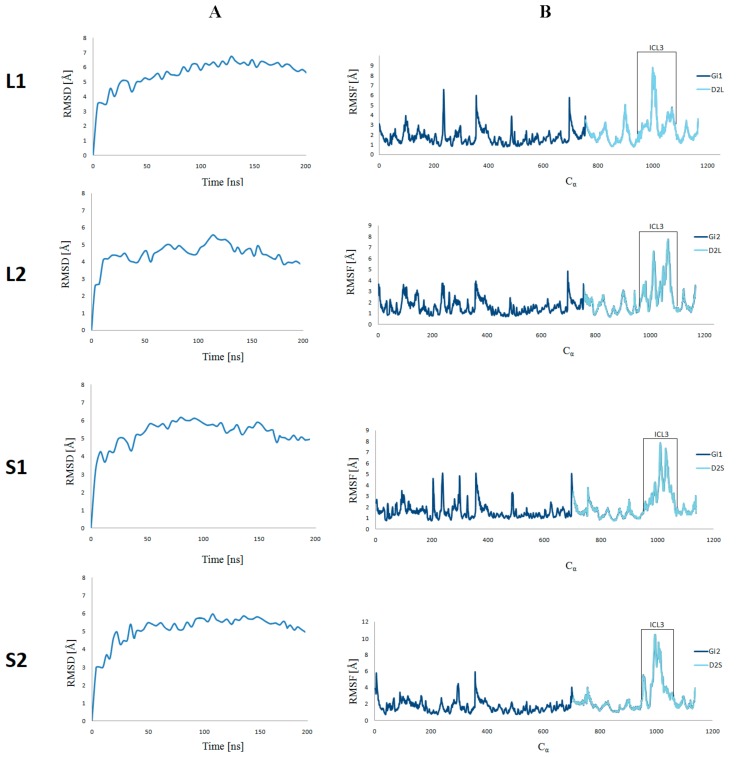
C_α_RMSD(**A**) and C_α_RMSF (**B**) of the four selected ligand–receptor complexes against the starting conformations during 200 ns molecular dynamics simulations.

For the preliminary MD simulations, 16 best-scored models (four from each population) in complex with dopamine were selected. Each ligand–receptor complex was immersed in a native-like membrane and subjected to minimization, equilibration, and production MD simulations of 200 ns. RMSD (root-mean-square deviation), RMSF (root-mean-square-fluctuation), and the protein-ligand short-range Lennard-Jones and Coulombic interaction energy analyses were performed. Figure 3 shows plots of four selected systems that were subjected to further processing. RMSD and RMSF values for the C_α_ atoms were calculated using Gromacs tools for preliminary 200 ns simulations to check for the stability of the models. The average RMSD values were: L1—4.76 Å, L2—4.09 Å, S1—4.97 Å, S2—4.56 Å, and RMSF: 1.95 Å, 1.80 Å, 1.79 Å, 2.00 Å, respectively. As expected, the ICL3 regions are characterized by the highest RMSF values compared to all other regions and they are the most flexible parts of the systems. The selected complexes were subjected to the production phase of molecular dynamics (1 µs) in three replicas each. Moreover, we simulated four corresponding systems without dopamine for comparison.

#### 2.3.1. Influence of the Loop Length on the Interaction of the Receptor with the G_i_ Protein Subtypes

**Figure 4:**
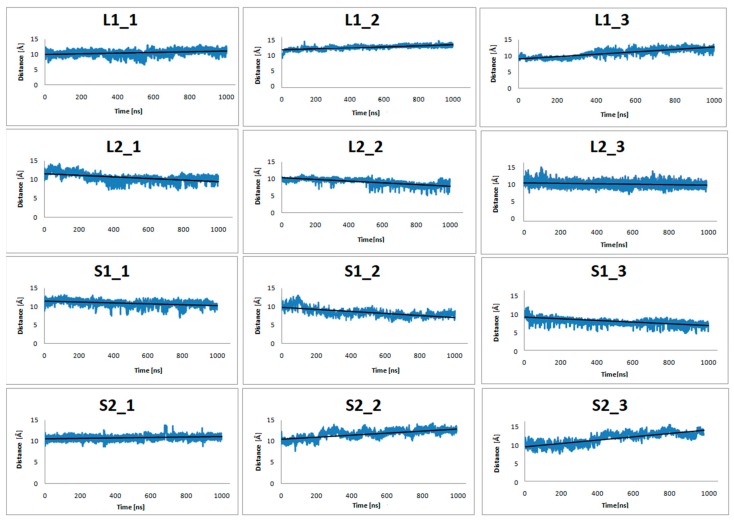
The distance of center-of-mass (COM) of Y7.53, L2.46, I3.46, T5.54, and V6.40 from the dopamine receptor and D351, C352, G353, L354, and F355from the α5-G_α_ protein for three replicas of the studied complexes (with dopamine bond) during 1 μs molecular dynamics (MD) simulations.

**Figure 5:**
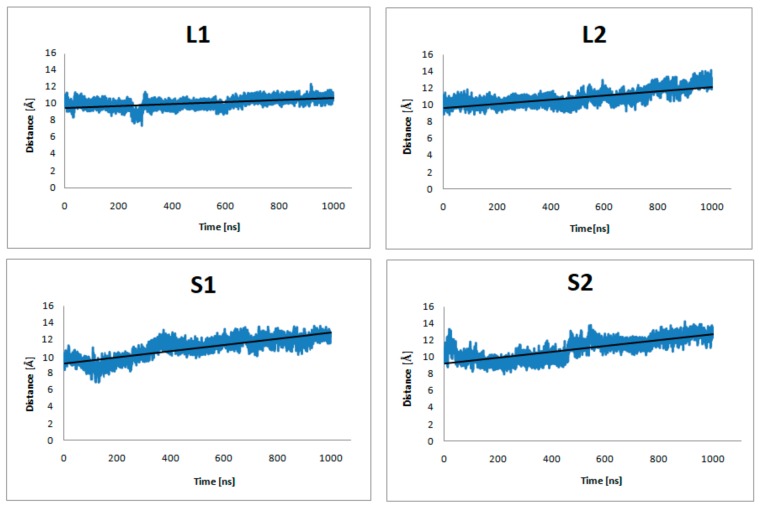
The distance of center-of-mass (COM) of Y7.53, L2.46, I3.46, T5.54, and V6.40 from the dopamine receptor and D351, C352, G353, L354, and F355 from the α5-G_α_ protein of the studied complexes (no dopamine bond) during 1 μs MD simulations.

Figure 4 shows plots of the center-of-mass (COM) distances between the C_α_ atoms of the receptor residues: Y7.53 in highly conserved NPxxY motif, L2.46, I3.46, T5.54, V6.40, and the C_α_ atoms of the α5-G_α_(C-terminus) G protein residues: D351, C352, G353, L354, F355 (in both G_i_ protein subtypes) during 1 µs MD simulations. In order to illustrate the tendency to decrease or increase the distance, the best fit line of all points was drawn. Figure 4 clearly shows that the distance between the studied residues decreases for the L2 and S1complexes. In contrast, for the L1 and S2 complexes, the studied distance increases. As the validation of these results, the same distance was measured for the systems without dopamine (Figure 5) and it increased in all cases. These results suggest the preferential coupling of the D_2L_ isoform with G_i2_ protein and D_2S_ with G_i1._

**Figure 6:**
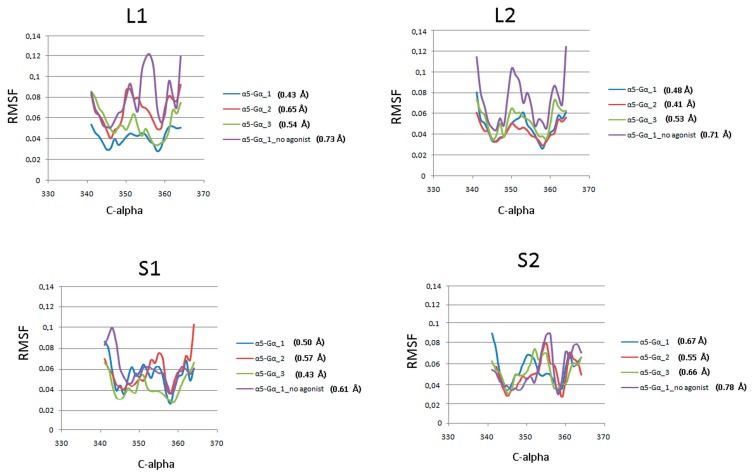
C_α_RMSF of the α5-G_α_ protein against the starting conformations during 1 µs molecular dynamics simulations. Each plot shows scores for three replicas and a receptor without agonist with the average value of RMSF.

Figure 6 presents plots for α5-G_α_ protein fluctuations from the entire simulation. The average values for S2 indicate greater fluctuations in this region compared to other complexes. In the case of L2, the average fluctuation values show that the studied region is more stable during 1 µs molecular dynamics simulations. To approximate the interaction between G protein and D_2_ receptor, the energy of interaction was calculated with the Gromacs tools. Table 2 shows the values of average short-range Lennard-Jones potential energy for all replicas and receptors without dopamine.

Furthermore, in order to investigate the interactions between G proteins and dopamine D_2_ receptor isoforms, the distance matrices consisting of the distances between residue pairs were calculated with gromacs tools (Supplementary Information, Figure S1). The last 200 ns of simulations were taken into account. On the distance map, points corresponding to the length from 0–3 Å are marked as black dots. Interactions between α5-G_α_ protein and D_2_ receptor residues are surrounded by a circle. The role of M6.36 is worth noting as it appears on all the maps except for simulations without agonist and all S2 simulations. In the case of S2 F7.56 appears in all simulations with the agonist. R6.58 appears in all simulations of L2 and S1. Moreover, the role of P139 of ICL2 is also apparent as it does not appear only for S1. In this case, R3.50 of DRY motif seems to play a role. It can be concluded, that the performed analysis did not allow to indicate the residues from dopamine D_2_ receptor isoforms which are responsible for the selectivity of interactions with respective G_i_ protein subtypes as all the indicated residues are shared by both isoforms. Probably, the positions and/or conformations of identified residues is different in four investigated complexes which can explain their differentiated importance for interactions with G_i_ protein subtypes.

#### 2.3.2. Structural Dynamics and Conformational Changes of Helices and Switches

**Figure 7:**
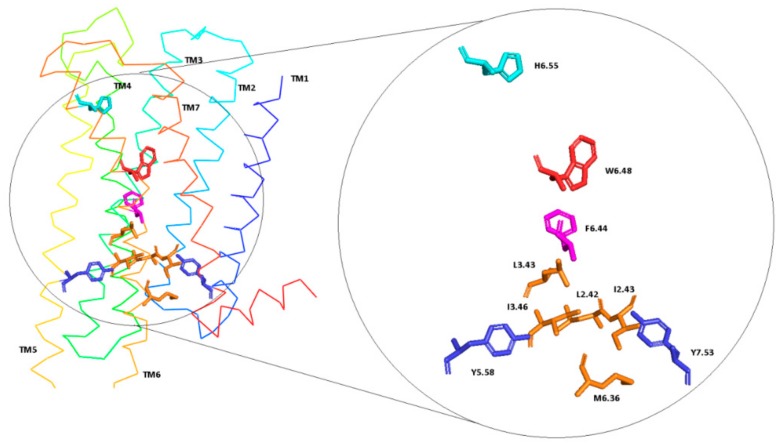
D_2_ receptor in inactive conformation with residues involved in molecular switches.

**Figure 8:**
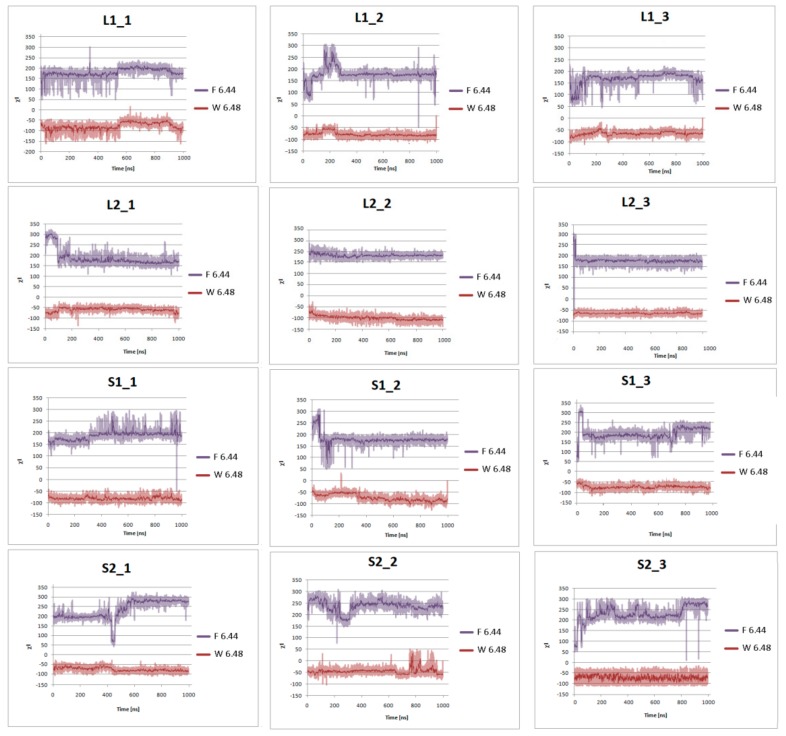
Side chain torsion angle changes of F6.44 and W6.48 for L1, L2, S1, and S2 receptors in complex with dopamine in three replicas during 1 μs molecular dynamics simulations.

**Figure 9:**
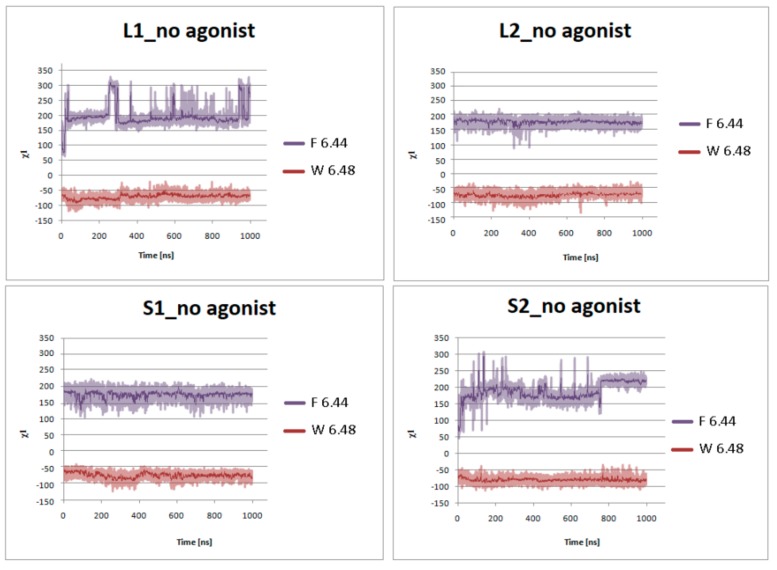
Side chain torsion angle changes of F6.44 and W6.48 for L1, L2, S1, and S2 receptors without dopamine during 1 μs molecular dynamics simulations.

**Figure 10:**
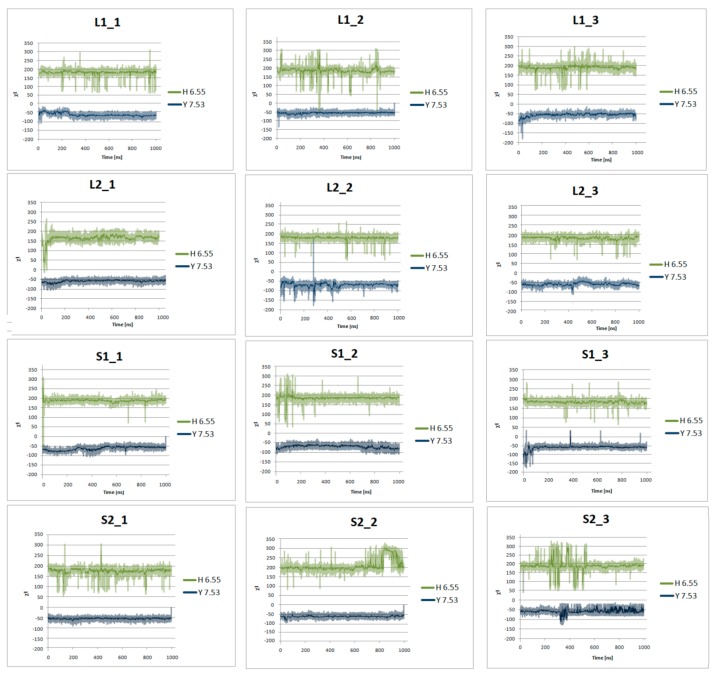
Side chain torsion angle changes of H6.55 and Y7.53 for L1, L2, S1, and S2 receptors in complex with dopamine in three replicas during 1μs molecular dynamics simulations.

**Figure 11:**
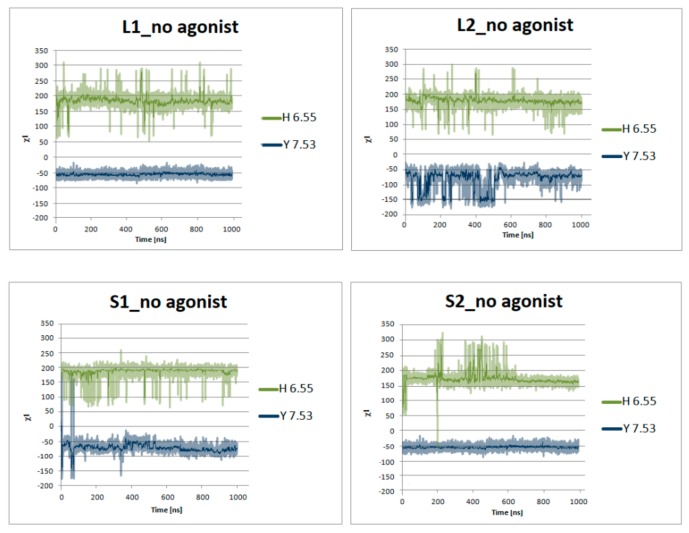
Side chain torsion angle changes of H6.55 and Y7.53 for L1, L2, S1, and S2 receptors without dopamine during 1 μs molecular dynamics simulations.

Figure 7 presents the helical region of the dopamine D_2_ receptor in inactive conformation with residues in molecular switches shown in detail: W6.48 of the CWxP motif (red), F6.44 of the PIF motif (pink), and H6.55 (cyan) in TM6 and Y7.53 (blue) of the NPxxY motif in TM7. Figure 8, Figure 9, Figure 10 and Figure 11 present the time evolution of the χ1side chain torsion angle of W6.48, F6.44, H6.55, and Y7.53 for systems with dopamine and without it. The studied dihedral angle of W6.48 is stabilized at about −60° in all simulations. The largest oscillations appear in all replicas of L1 as well as in the second and third replicas of S2. Residue F6.44 showed significant flexibility oscillating around +180° in all simulations for the receptor with agonist. In L2 with agonist, the investigated dihedral angle showed the lowest conformational flexibility. The highest oscillations appeared in all replicas of S2 and L1 in the initial parts of simulations. Residue Y7.53 showed rather low variations with values oscillating around −60°. In the case of L2 in the second replica with agonist, L2 without agonist and S1 without agonist, significant flexibility can be observed. Interestingly, for the χ1 of H6.55 remarkable differences were found. For L1 and S2 in all simulations with and without agonist, the dihedral shows large oscillations for most of the simulation time. In simulations of L2 and S1, this conformational change does not occur so often, although those regions are flexible, and the dihedral angle oscillated at around 180°.

**Figure 12:**
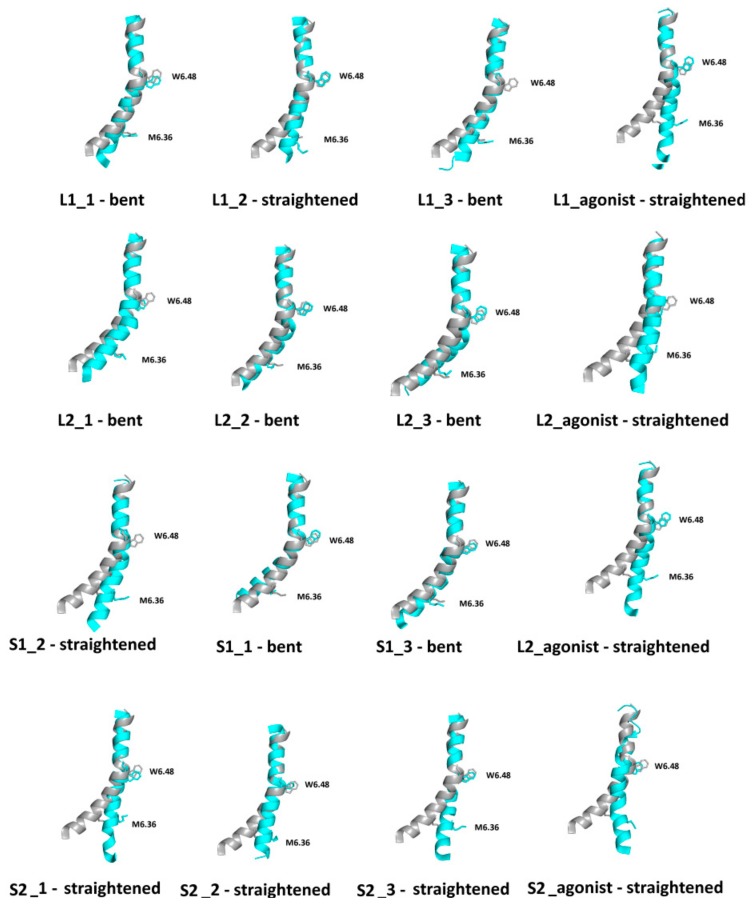
Superimposition of TM6 for initial simulation (grey) and TM6 (cyan) after 1 µs MD simulations with conformational state described below.

One of the hallmarks of GPCR active conformation is described by the transmission switch (former Trp rotamer toggle switch) [11,13]. In short, in all GPCR crystal structures with agonists, movements of TM5 and TM6 can be observed, including relocation of conserved residues Trp6.48 and Phe6.44 toward Pro5.50. It results in the bending of TM6 at the CWxP motif in active structures in contrast to straightened TM6 in inactive structures. Figure 12 shows TM6 conformational changes for the receptors with and without dopamine after 1 µs molecular dynamics simulations. L1 with an agonist showed slight differences between the starting conformation and the conformation after MD simulations. The difference appeared below the rotamer toggle switch, W6.48, where a slight bending in the two out of three replicas was observed. In the second replica and for the receptor without agonist, a tendency to straighten TM6 is evident. An interesting observation was found for L2 and S2 complexes. In the first case, significant helical bending can be seen below the W6.48 for all simulations with dopamine. For S2, all simulations showed a tendency to straighten TM6 and shift M6.36 into the protein interior. For S1, only in the one replica and the receptor without agonist TM6 clearly changed the spatial conformation. MD simulations also showed that, for receptors without dopamine, the tendency to straighten TM6 is notable. These observations further confirm the preferential coupling of the D_2S_ isoform with G_i1_ protein while D_2L_ isoform can be coupled mainly to the G_i2_ protein and also to the G_i1_ protein.

**Figure 13:**
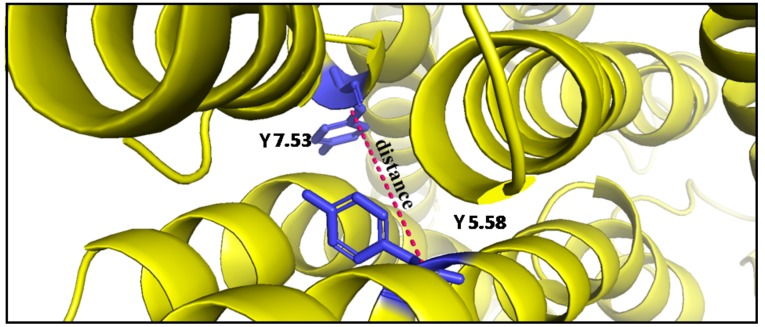
Distance between TM5 and TM7 (yellow) at the Cα atom of Y5.58 and Cα atom of Y7.53 (blue).

The distance between TM5 and TM7 at Cα atoms of Y5.58 and Y7.53 was also examined (Figure 13). Table 3 shows the distances for receptors after 1 µs MD simulations. It is clearly seen that the distances between Y5.58 and Y7.53 for L1 and S2 were significantly higher than for L2 and S1. In comparison to the inactive state D_2_ crystal structure (PDB: 6CM4 [19]: 19.4 Å), the Y5.58–Y7.53 distance in the S2 structure showed the highest values, indicating a structural similarity with the inactive state of the receptor, while L2 showed the smallest distance value in all simulations further confirming preferential G_i_ subtype protein coupling.

**Figure 14:**
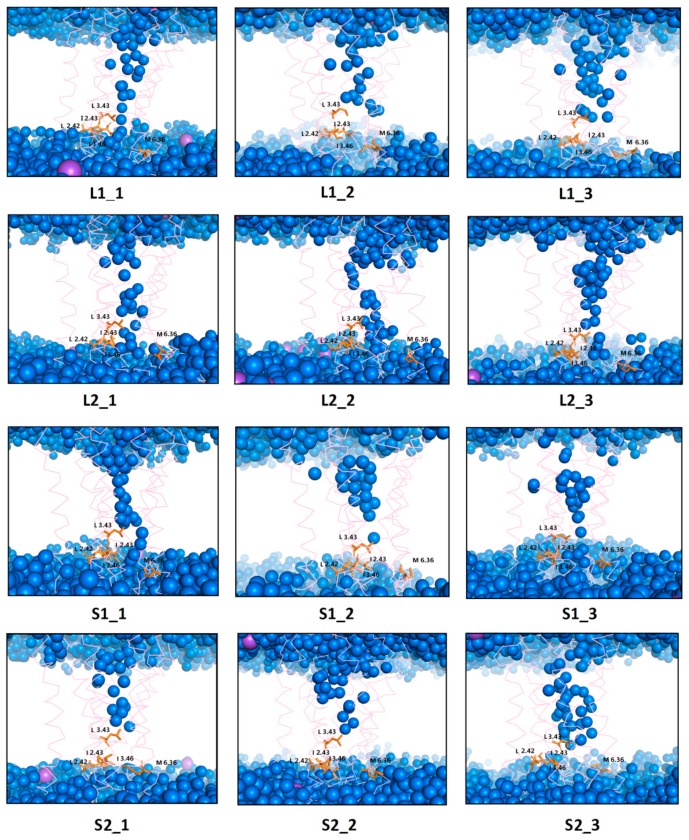
Internal water channel with marked residues (orange) forming a hydrophobic barrier for receptors with dopamine after 1 µs MD simulations. The helices are marked as pink springs and the sodium ions as a purple sphere.

**Figure 15:**
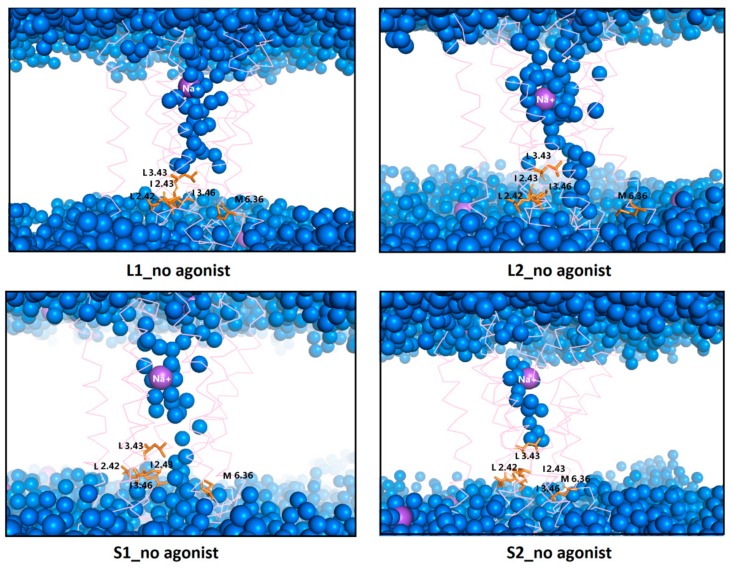
Internal water channel with marked residues (orange) forming a hydrophobic barrier for receptors without dopamine after 1 µs MD simulations. The helices aremarked as pink springs and the sodium ions as a purple sphere.

It is also well-known that activation of GPCRs correlates with the formation of a continuous internal water pathway as a hydrophobic layer of amino acid residues next to the characteristic NPxxY motif forms a gate that opens to form a continuous water channel only upon receptor activation [26]. Rearrangement of TM3, TM6, and TM7 affects the formation of the water channel visible in Figure 14 and Figure 15. The water channel remained open for all replicas of L2 with dopamine, one replica of L1 with dopamine, and two replicas of S1 with dopamine. For S2, in all replicas, water molecules were blocked at the hydrophobic barrier consisting of five residues: L2.42, I2.43, L3.43, I3.46, and M6.36 (orange in Figure 7). This situation also happened in the case of L1 in two replicas where TM5, TM6, and TM7 conformation changes were not clearly visible. This data is consistent with the results described above. Surprisingly, for L2 and S1 without dopamine, the water channel remained open during the MD simulations as well as for L2 with dopamine for all replicas and S1 in two replicas, which indicates a slower deactivation compared to L1 and S2. In simulations without dopamine, sodium ion (purple in Figure 15) diffused from the solvent layer into the deep orthosteric pocket of the receptor, which is consistent with the results described above.

**Figure 16:**
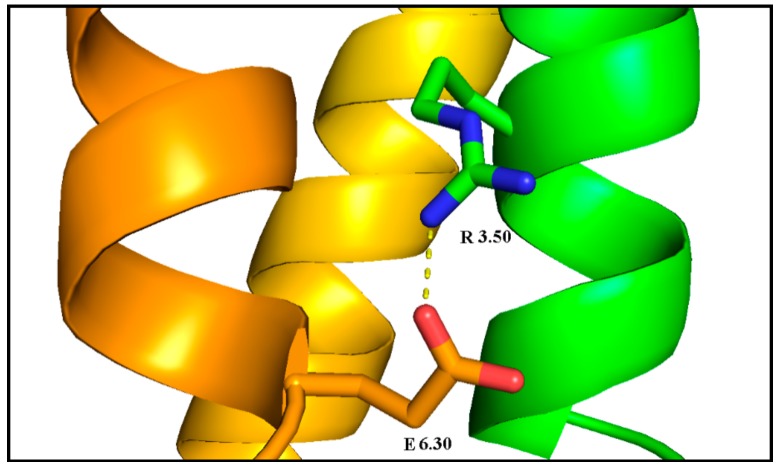
Ionic lock of inactive the dopamine D_2_ receptor (PDB ID: 6CM4): the lock between R3.50 (green with blue nitrogen atoms) of the DRY motif and E6.30 (orange with red oxygen atoms).

Another important feature that distinguished receptor conformational changes after 1 µs MD simulations was the distance between the R3.50 of the DRY motif and E6.30, forming the interhelical ionic lock involving TM3 and TM6. Because of the fact that receptor models were constructed on the active 3SN6 template, the distance values, given in Table 4, are much higher than in the inactive crystal structure of the dopamine D_2_ receptor (3.0 Å) showed in Figure 16. However, differences in distances are notable. The smallest distance appeared in all S2 replicas. All receptors without dopamine showed lower values than their counterparts with dopamine. The highest values(>20 Å) appeared for all replicas of L2 and S1 supporting our previous observations.

Referring to the literature data indicating that R233 from the ICL3 of D_2S_ isoform is important for interaction with G protein [8], we also investigated the interactions between the α subunit of G protein and R233 in ICL3 of both isoforms. Table 5 presents the average distances between the side chain COM of D309, L310, and K312 in G_iα1_ or K307, D310, and L311 in G_iα2_ and side chain COM R233 in ICL3 during the last 100 ns of simulations. In the case of L2 and S2, the smallest distance is observed. Moreover, in two replicas of L2 and one replica of S2, the hydrogen bond in the last step of the simulation is formed between D310 from the G protein and R233 from the ICL3 of the receptor.

### 2.4. Principal Component Analysis

For additional support, MD simulations were analyzed by principal component analysis (PCA)for all the trajectories concatenated in four populations separately for L1, L2, S1, and S2 with their counterparts without dopamine. The conformational changes of TM6 and TM5 in combination with TM7 were considered by non-mass-weightened analysis. The projections were for the last 100 ns of the MD simulations. Covariance matrices of individual subspaces were compared, using the Gromacs tools, and are given in Table 6. The overlap is 1 if matrices are identical. It is 0 when the sampled subspaces are totally orthogonal [27]. Our result allows the comparison of subspaces with a slight deviation only.

**Figure 17:**
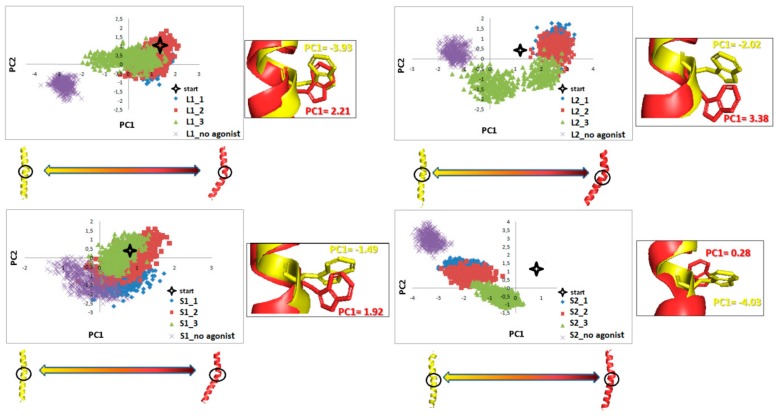
Motion of the protein in phase space along the first two principal componentsfor TM6 in the last 100 ns of dynamics. The yellow and red cartoons correspond to the PC1 values and show extreme projections along PC1.

Figure 17 shows the conformational space of TM6 explored in MD simulations, described by PCA projection of the motion of the TM6 (fragments covering the last 100 ns of simulations are presented to improve clarity). Clusters for receptors without agonists are negative along PC1, which corresponds to straightening the TM6 at W6.48. For L1 and S2, there are significant deviations from the start conformation. L2 shows a shift toward positive values for PC1 (bending TM6 at W6.48) while S2 takes negative values. We can see that TM6 of S2 adopts a similar conformation state as TM6 for the receptor without an agonist. The changes for L1 and S1 show slight shifts compared to the starting structure, which is also shown in Figure 12 with the final TM6 conformations.

**Figure 18:**
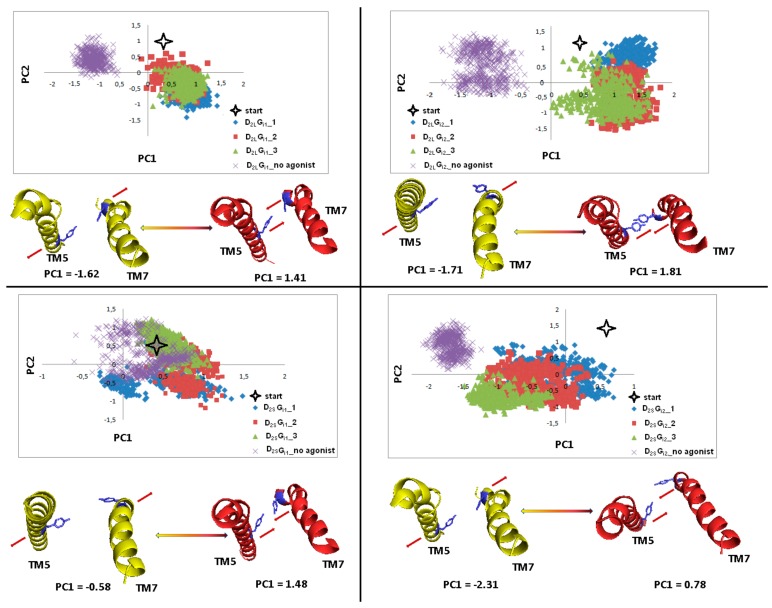
Motion of the protein in phase space along the first two principal components for TM5 and TM7 in the part including Y5.58 and Y7.53 (blue). The yellow and red cartoons, with the red arrows describing the tendency to the extreme conformations, correspond to the PC1 values and show extreme projections along PC1.

Figure 18 shows the motion of the protein in phase space along PC1 and PC2 values for TM5 in combination with TM7 at Y5.58 and Y7.53. Clusters for receptors without an agonist for L1, L2, and S2 are negative along PC1. It is significant that clusters of S2 with agonist have mostly negative values, which means adopting conformation similar to the receptor without dopamine. The changes for S1 show slight shifts compared to the starting structure. Thus, PCA results support our conclusions described above.

## 3. Discussion

The aim of our work was to construct full models of the dopamine D_2_ receptor D_2S_ and D_2L_ isoforms in complex with a natural agonist, dopamine, and to study the coupling of these isoforms with G_i1_ and G_i2_ proteins, as the experimental data about D_2S_ and D_2L_ isoforms and G_i_ protein subtype preference remains unclear [6,8]. Although models of the dopamine D_2_ receptor in active conformation with or without the respective G protein are already available in the literature [28,29,30,31], this is, to our best knowledge, the first time full D_2S_ and D_2L_ isoforms, including ICL3 loop, have been modeled. Modeling of ICL3 in the case of GPCRs with a long ICL3 is a challenge as there are no templates for this highly flexible protein fragment. However, intracellular loops, in particular ICL3, play an important role in receptor activation due to their interaction with G proteins and the influence on G protein preferential coupling [32,33,34]. Recently, a full model of human serotonin 5-HT_2A_ receptor has been reported as an example of this challenging loop modeling [15].

Before any further investigations could be performed, an in silico model needs to be properly validated. The protocol for the construction of a homology model of the dopamine D_2_ receptor in active conformation in complex with G protein using multiple templates is based on our earlier experience with the modeling of the μ opioid receptor in active conformation [35,36,37,38]. This protocol turned out to be successful as the comparison of the μ opioid receptor model with the crystal structure revealed the C_α_RMSD of whole structures of 2.60 Å, while removal of the most disordered regions (N-terminus, C-terminus, ICL3) decreased RMSD to 1.91 Å, below the crystal resolution (2.10 Å) [37]. The short and long ICL3 loop were modeled based on the predicted secondary structure and the interactions of these loops with respective regions of G proteins are in accordance with experimental data [9]. The final models were selected from populations of 100 models each and assessed using internal Modeller scoring functions as well as validated applying widely used tools for this purpose. According to all the studied factors, the models were characterized by good quality. The docking of dopamine to the models, in agreement with available literature data, further confirms the correctness of the models. Next, we performed preliminary molecular dynamics studies of 16 models (four models of each type) to select, after 200 ns of simulations, the best-scored four systems for further investigations. Finally, our simulations were performed in a native-like membrane environment, which allows us to reproduce correctly molecular events concerning GPCR functioning and to follow subtle aspects of these receptors’ early activation at the molecular level [39,40].

As there are no reports about structural aspects of the full-length dopamine D_2_ receptor concerning its interactions with respective G proteins, we investigated how the length of the ICL3 loop affects the interactions with the G_i1_ and G_i2_ proteins and receptor activation processes. It turned out that differences in loop length contribute to the different behavior of the receptor when it binds to a specific G protein. The increasing distance between the intracellular parts of dopamine receptor D_2L_ isoform transmembrane helices, and α5-G_i1_, the binding region of G protein, indicates that G_i1_ protein is sliding out of the receptor-binding surface and the signal transduction started by agonist binding is stopped. The corresponding distance for the D_2L_ and G_i2_ complexes decreases slightly, which may indicate that the active state is maintained. Simulations without dopamine showed that the described protein regions move away from each other, which may also indicate the deactivation process and which constitutes an additional validation of our simulations. In the simulations for D_2S_ isoform, it can be seen that the D_2S_ receptor moves away from the α5-G_i2_ protein. The decrease in the distance between α5-G_α_ helix and the intracellular parts of receptor transmembrane helices for L2 and S1 complexes in all replicas suggest preferential coupling of the D_2L_ isoform with G_i2_ protein and D_2S_ isoform with G_i1_ protein, which is in agreement with experimental data obtained by Montmayeur et al. [6] and Grünewald et al. [7].Thus, the length of the ICL3 loop of dopamine D_2_ receptor isoforms governs coupling with the respective G_i_ protein subtypes, which can be of importance to explain different in vivo roles of these isoforms. Experimental studies confirmed the importance of ICL3 loop N- and C-termini for G protein coupling [41,42].

Many studies have shown that GPCRs share a set of residues called molecular switches that are involved in receptor activation and signal transduction [26,43]. The rearrangement of the hydrophobic residues in TM2, TM3, and TM6 helices are involved in signal propagation [44]. In the inactive state of dopamine, D_2_ receptor residues L2.42, I2.43, L3.43, I3.46, and M6.36 (orange on Figure 7) constitute a hydrophobic pocket which prevents the creation of hydrogen bond network involved in the interaction with G protein. Our molecular dynamics simulations show that in the case of systems where the distance to respective G proteins is reduced, the hydrophobic barrier is broken and the flow of water molecules in the water channel is enabled, which is characteristic for the active state of GPCRs [45,46]. Interestingly, some systems without dopamine also maintained an open hydrophobic barrier, which may indicate a slow receptor deactivation, and further support the assumption that breakdown of the intra-receptor water chain in many dopamine-bound L1 and S2 complexes was caused rather by non-compatible allosteric signals from binding partners than by chance. Moreover, PCA results show that one of those systems—S1—maintains many similarities to agonist-bound systems, which may indicate that in this particular combination the allosteric effect of G protein coupling on the receptor conformation is considerable. We also examined the conformational changes of TM6 and found that its bending is correlated with the distance between TM5 and TM7 in the place of two highly conserved tyrosine residues Y5.58 and Y7.53.Systems with the tendency to straighten TM6 were also characterized by the largest distances between TM5 and TM7.The stronger bending leads to closer interaction between Y5.58 and Y7.53, which was suggested to stabilize the active state of the receptor via the water-mediated hydrogen bond [47,48].Our results regarding the conformation of TM6 further support preferential coupling of dopamine receptor isoforms with respective G_i_ protein subtypes.

We also examined the behavior of several microswitches, which are important for the GPCR activation process. We analyzed changes in the dihedral angle of the conserved W6.48 [43,49,50,51], F6.44 (called transmission switch [52,53,54]), Y7.53 of NPxxY motif [32,55], and H6.55 (a crucial residue for dopamine D_2_ receptor activation [56,57]). It was found that H6.55 is characterized by a high degree of conformational flexibility. We also investigated the behavior of the residues forming ionic lock between R3.50 and E6.30 and noticed the correlation between the ionic lock distance and other hallmarks of receptor active or inactive state. The lowest values were observed for complexes without dopamine and for the D_2S_G_i2_, so we can assume that these models tend to adopt inactive conformation of the receptor.

## 4. Materials and Methods

### 4.1. Receptor Model Construction

The sequences of the human D_2L_ and D_2S_receptors, G_αi1_ and G_αi2_ proteins were obtained in FASTA format [58] from the UniProt database (https://www.uniprot.org/).The crystal structure of the human β_2_adrenergic receptor complexed with a heterotrimeric G_s_protein (PDB ID: 3SN6 [18]) was used as a template for the helix bundle for homology modelingof D_2L_ and D_2S_ receptor isoforms in the active conformation as well as the template for G protein. In addition, the crystal structures of human dopamine D_2_, D_3_, and D_4_ receptors (PDB IDs: 6CM4 [19], 3PBL [20], and 5WIU [21], respectively) were used as templates for the extracellular loops. Multiple sequence alignment was carried out with MUSCLE (Multiple Sequence Comparison by Long-Expectation) [59].

The homology models of D_2L_ and D_2S_receptors in active conformation in complex with the respective G proteins were built using Modeller v. 9.19 (Andrej Ŝali, San Francisco, USA) [60]. The models of the D_2L_ and D_2S_ ICL3 loops were generated with Yasara software with restrictions imposed on secondary structure predicted by PredictProtein [22] online server (https://www.predictprotein.org/). Ten models were created in each of the four model populations (L1, L2, S1, S2) differing in the loop conformation. The most probable loop models for the long and short loops were selected on the basis of their potential interactions with G proteins and favorable orientation in the intracellular area (with no overlap with the membrane).

Modeller was applied to incorporate the loops to the four final populations of models (100 models each). The final populations of models were evaluated based on their Discrete Optimized Protein Energy (DOPE) profiles obtained from Modeller (Twenty models with the lowest DOPE values were selected from each population. They were validated using Verify3D (https://servicesn.mbi.ucla.edu/Verify3D/) [24], ERRAT (https://servicesn.mbi.ucla.edu/ERRAT/) [25], and ProCheck (https://servicesn.mbi.ucla.edu/PROCHECK/) [23]. Four best-scored models from each population were selected for further studies.

### 4.2. Molecular Docking

The structure of orthosteric ligand, dopamine, was modeled using the Hartree–Fock approach and 6-31G* basis set of Spartan v. 10 VI.0.1(Wavefunction, Inc., Irvine, California, USA) [61]. MolegroVirtual Docker 6.0 software (Molexus IVS, Odder, Denmark) [62] was used for docking simulations of flexible ligand dopamine into the rigid receptor models. The actual docking simulations were performed using the following settings: number of runs = 100; maximal number of iterations = 10,000; maximal number of poses = 50; and the poses representing the lowest value of the scoring function (MolDockScore) were further analyzed as previously reported [63,64]. The most probable docking pose of dopamine was selected from the poses where a protonatable nitrogen atom of dopamine formed an electrostatic interaction with the conserved aspartate from the third transmembrane helix, D3.32, taking into account available literature data on other interactions between dopamine and D_2_ receptor [65].

### 4.3. Molecular Dynamics

Sixteen final ligand–receptor complexes were subjected to molecular dynamics with Gromacs version 2018.4 [66] in native-like conditions. The membrane environment for the complexes was prepared using the Charmm-GUI Membrane Builder server [67]. The complexes were immersed in an asymmetric membrane consisting of nine types of lipids in the proportions appropriate for membrane rafts [68] containing cholesterol, sphingomyelin, DOPE, DOPC, DOPS, PLPC, POPC, POPE, POPG, and aqueous phase: TIP3P water molecules with 0.15 M NaCl. A 3SN6 crystal structure was used as the receptor orientation template in a membrane. An Amber03 force field [69] was used for receptors, Slipids (Stockholm lipids) [70] for the membrane, and General Amber Force Field (GAFF) [71] for ligands. EPS charges were obtained by RESP ESP charge Derive Server [72] and processed with the ACPYPE server [73] to gain ligand topologies. The properly protonated receptor structures were obtained from the H++ server [74]. A template receptor (β_2_ adrenergic receptor in complex with G_s_ protein) in a membrane was first minimized using 500 steps. Then, it was equilibrated in 1 ns NPT simulations using the Berendsen barostat to control volume fluctuations, followed by 5 ns NVT simulations and 10 ns NPT simulations using the Parrinello–Rahman barostat. The template receptor in the equilibrated system was changed to 16 dopamine–receptor (4 × L1, 4 × L2, 4 × S1, and 4 × S2). Each system was again minimized, and equilibrated under 1 ns NVT and 5 ns NPT as above with protein and ligand position restrains (force constants of 10,000 kJ mol^−1^ nm^−2^) on the heavy atoms. The molecular dynamics simulations of 16 systems were performed for 200 ns using a time-step of 2 fs. Four systems (one of each type: L1, L2, S1, and S2) were selected based on the comparison of RMSD, RMSF, and energy of dopamine–receptor interactions (the protein–ligand short-range Lennard-Jones and Coulombic interaction energy). These systems were subjected to the production phase of molecular dynamics (1 µs) in three replicas each. As a reference, these systems without dopamine were also simulated. Standard Gromacs tools were used for the analysis of the results.

## 5. Conclusions

In summary, we used for the first time in silico approaches to construct models of full D_2S_ and D_2L_ dopamine D_2_ receptor isoforms and studied their coupling with G_i_ protein subtypes. Our results indicate the preferential coupling of the D_2L_ isoform with G_i2_ protein and D_2S_ isoform with G_i1_ protein, which is in accordance with experimental data. The G_i_ protein subtype preference is further supported by different hallmarks of receptor active state, including conformation of microswitches, the conformation of TM6, and the formation of the water channel. The results in this study give mechanistic insight in the interplay between isoforms of dopamine D_2_ receptors and G_i_ proteins subtypes, which is important to understand signaling by these receptors and their mediation by pharmaceuticals—in particular, psychotic and antipsychotic agents.

## Figures and Tables

**Table 1:** Results of validation of the best-scored receptor-G protein complexes models.

Web Server	Parameters	Scores															
		L1				L2				S1				S2			
ProCheck	Favored region (%)	91.2	92.4	92.7	92.1	94.3	93.5	93.7	91.8	94.5	94.3	92.1	94.6	94.2	91.4	94.0	93.3
	Allowed region (%)	7.1	6.9	6.2	6.8	4.6	5.0	4.3	7.8	4.3	4.2	6.7	4.4	3.4	7.1	4.7	5.5
	Outlier region (%)	1.7	0.7	1.1	1.1	1.1	1.5	2.0	0.4	1.2	1.5	1.2	1.0	2.4	1.5	1.3	1.2
Verify 3D	Averaged 3D–1D score ≥0.2 (%)	82.4	83.1	83.2	83.4	84.2	83.4	83.9	83.4	84.7	83.2	82.9	84.3	83.3	82.4	83.9	83.4
ERRAT	Overall quality factor	90.8	91.3	91.1	91.4	94.2	93.2	93.6	92.3	93.3	93.4	91.7	92.6	94.7	90.9	92.4	92.3

**Table 2:** Values in (kJ/mol) of the average short-range Lennard-Jones potential energy of 1 µs MD simulations for the COM of C-terminal part of α5-G_α_: G353, L354, F355 and residues from the DRY-motif of TM3: D3.49, R3.50, from IL2: M140, Y142 and TM6: K6.28, M6.36 for all replicas and receptor without dopamine.

Complex	Replica			
	1	2	3	No Agonist
L1	−850.34 kJ/mol	−824.64 kJ/mol	−867.54 kJ/mol	−734.98 kJ/mol
L2	−1134.79 kJ/mol	−914.32 kJ/mol	−1156.62 kJ/mol	−847.72 kJ/mol
S1	−919.73 kJ/mol	−962.38 kJ/mol	−898.54 kJ/mol	−805.22 kJ/mol
S2	−766.54 kJ/mol	−685.39 kJ/mol	−772.47 kJ/mol	−550.27 kJ/mol

**Table 3:** Distance values for receptors between Y5.58 and Y7.53 1 µs MD simulations.

Complex	Replica			
	1	2	3	No Agonist
L1	13.3 ± 0.4 Å	14.1 ± 0.3 Å	14.2 ±0.5 Å	16.1 ±0.4 Å
L2	10.1 ± 0.5 Å	8.5 ± 0.3 Å	11.7 ± 0.4 Å	15.1 ± 0.3 Å
S1	12.3 ± 0.3 Å	12.6 ± 0.5 Å	11.4 ± 0.4 Å	15.6 ±0.3 Å
S2	18.5 ± 0.3 Å	16.2 ± 0.3 Å	17.9 ±0.2 Å	19.1 ±0.3 Å

**Table 4:** Distance values for receptors between R3.50 and E6.30 after 1 µs MD simulations.

Complex	Replica			
	1	2	3	No Agonist
L1	18.1 ± 0.2 Å	18.8 ± 0.2 Å	19.5 ± 0.4 Å	18.0 ± 0.3 Å
L2	21.7 ± 0.4 Å	22.1 + 0.3 Å	20.1 ± 0.5 Å	19.0 + 0.3 Å
S1	21.9 ± 0.2 Å	22.0 ± 0.4 Å	21.9 ± 0.2 Å	14.7 ± 0.3 Å
S2	16.8 ± 0.3 Å	14.4 ± 0.3 Å	18.8 ± 0.2 Å	13.6 ± 0.4 Å

**Table 5:** The average distances between the side chain COM of D309, L310, and K312 in G_α1_ or K307, D310, and L311 in G_α2_ and side chain COM R233 in ICL3 of D_2S_ or D_2L_ during the last 100 ns of the simulations.

Complex	Distance		
	D309	L310	K312
**L1**	12.1 ±0.2 Å	13.6 ±0.3 Å	11.1 ±0.2 Å
**S1**	13.1 ±0.3 Å	11.8 ±0.3 Å	10.8 ±0.2 Å
	**K307**	**D310**	**L311**
**L2**	7.5 ±0.3 Å	5.9 ±0.3 Å	8.4 ±0.2 Å
**S2**	10.0 ±0.4 Å	8.1 ±0.2 Å	10.6 ±0.2 Å

**Table 6:** Values of the overlap of the covariance matrices.

Complex	L1	L2	S1	S2
**L1**	-	0.758	0.731	0.698
**L2**	0.758	-	0.659	0.686
**S1**	0.731	0.659	-	0.761
**S2**	0.698	0.686	0.761	-

## Supplementary Materials

Supplementary materials can be found at https://www.mdpi.com/1422-0067/21/2/436/s1.

## Abbreviations

L1	D_2LONG_ receptor in complex with G_i1_
L2	D_2LONG_ receptor in complex with G_i2_
S1	D_2SHORT_ receptor in complex with G_i1_
S2	D_2SHORT_ receptor in complex with G_i2_
DOPE	Discrete Optimized Protein Energy
ECL	Extracellular loop
GPCRs	G protein coupled receptors
ICL	Intracellular loop
MD	Molecular dynamics
PCA	Principal Component Analysis
RMSD	Root-mean-square deviation
RMSF	Root-mean-square fluctuation
TM	Transmembrane
