# Supplementary material for: Preferential Coupling of Dopamine D2S and D2L Receptor Isoforms with Gi1 and Gi2 Proteins—In Silico Study
Source: Int J Mol Sci. 2020 Jan 9;21(2):436. doi: 10.3390/ijms21020436 (PMC7013695; doi:10.3390/ijms21020436)
Supplement: Supplementary file 1 [file ijms-21-00436-s001.pdf]

Supplementary Information

Preferential coupling of dopamine D_{2S} and D_{2L} receptor isoforms with G_{i1} and G_{i2} proteins – *in silico* study

Justyna Żuk[1] , Damian Bartuzi[1] , Dariusz Matosiuk[1] and Agnieszka A. Kaczor[1,2*]

1 Department of Synthesis and Chemical Technology of Pharmaceutical Substances with Computer Modeling Laboratory, Faculty of Pharmacy, Medical University, 4A Chodzki St., PL-20093 Lublin, Poland

2 School of Pharmacy, University of Eastern Finland, Yliopistonranta 1, P.O. Box 1627, FI-70211 Kuopio, Finland

* Correspondence: agnieszka.kaczor@umlub.pl

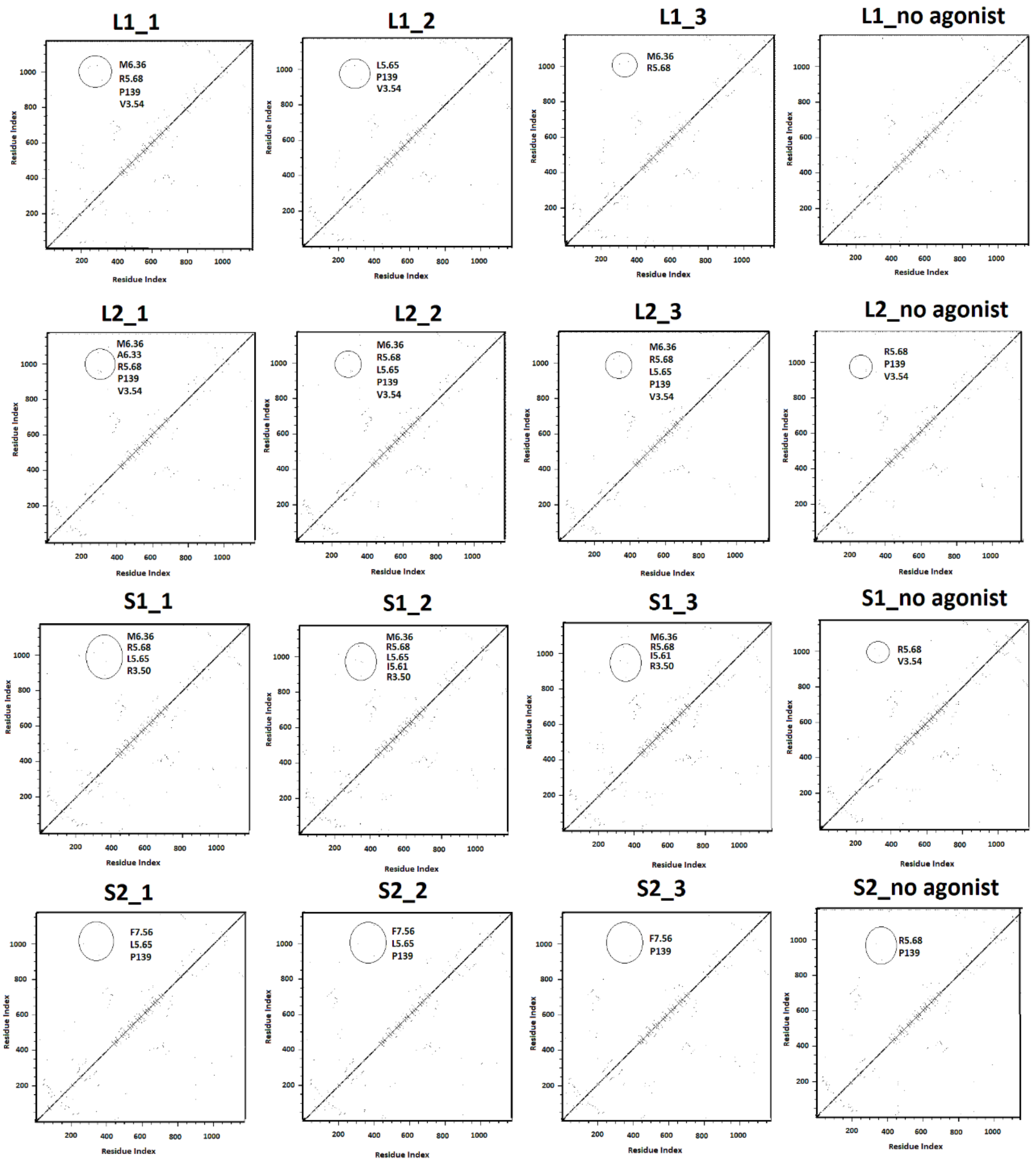

Figure S1. Distance maps of interactions of dopamine D_2 receptor isoforms with G_i protein subtypes.
